# A glance at transient hyperammonemia of the newborn: Pathophysiology, diagnosis, and treatment: A review

**DOI:** 10.1097/MD.0000000000031796

**Published:** 2022-12-02

**Authors:** Beibei Ni, Miao Qin, Jun Zhao, Qie Guo

**Affiliations:** a Department of Clinical Pharmacy, The Affiliated Hospital of Qingdao University, Qingdao, China; b Department of Neonatology, The Affiliated Hospital of Qingdao University, Qingdao, China.

**Keywords:** ammonia, CRRT, CVVH, CVVHDF, hyperammonemia, transient hyperammonemia of the newborn

## Abstract

Hyperammonemia is the excessive accumulation of ammonia in the blood, and is usually defined as a plasma level above 100 µmol/L in neonates or above 50 µmol/L in term infants, children, and adolescents. Patients with hyperammonemia usually experience life-threatening neuropsychiatric symptoms, especially newborns. It is routinely caused by inherited metabolic diseases and also by acquired disorders, such as liver failure, portosystemic shunting, gastrointestinal hemorrhage, ureterosigmoidostomy, renal tubular acidosis, hypoxic ischemic encephalopathy, infections with urea-metabolizing organisms, and some drugs. Transient hyperammonemia of the newborn (THAN) is a special type of hyperammonemia acknowledged in the field of metabolic disease as an inwell-defined or well-understood entity, which can be diagnosed only after the exclusion of genetic and acquired causes of hyperammonemia. Although the prognosis for THAN is good, timely identification and treatment are essential. Currently, THAN is underdiagnosed and much less is mentioned for early diagnosis and vigorous treatment. Herein, we present common themes that emerge from the pathogenesis, diagnosis, and management of THAN, based on current evidence. When a newborn presents with sepsis, intracranial hemorrhage, or asphyxia that cannot explain coma and seizures, doctors should always keep this disease in mind.

## 1. Introduction

Hyperammonemia is the excessive accumulation of ammonia in the blood, which is an acute life-threatening condition that leads to severe neurological impairment and cerebral edema.^[1]^ Hyperammonemia is routinely caused by inherited metabolic diseases, such as congenital defects in the urea cycle, classical organic acids, and defects in mitochondrial fatty acid oxidation.^[2]^ It can also be caused by acquired disorders such as liver failure,portosystemic shunting, gastrointestinal hemorrhage, ureterosigmoidostomy, renal tubular acidosis, hypoxic ischemic encephalopathy, and infections with urea-metabolizing organisms, in addition to exposure to drugs such as valproate, L-asparaginase, ribavirin, 5-flurouracil, and teprenone.^[3–5]^ Transient hyperammonemia of the newborn (THAN) is a disease whose incidence is seriously underestimated and manifests as a hyperammonemic coma in premature infants. THAN can only be diagnosed after the exclusion of genetic and acquired causes of hyperammonemia. THAN has been acknowledged in the field of metabolic disease as an not well-defined or well-understood entity since it was first described in 1978 by Ballard RA.^[6,7]^ Asymptomatic THAN is very common, occurring in > 50% of premature infants during the first 2 months of life.^[8]^ Symptomatic THAN is more prominent only when blood ammonia levels increase to frightening levels in comatosed patients.^[9]^ In neonates with severe THAN, lethargy, irritability, poor feeding, vomiting, hypotonia, tachypnea deterioration, acute encephalopathy, seizures, coma, and even death have been characterized.^[10]^ Herein, we review the pathogenesis, diagnosis, and treatment of THAN and provide new insights into its management.

## 2. Pathogenesis of THAN

Ammonia is a common constituent of body fluids. The primary source of ammonia production is bacterial protein metabolism, which produces colonic urease that converts dietary amino acids and urea into ammonia.^[11]^ Ammonia is produced in the intestinal tract, detoxified in the liver, and excreted through the kidneys. Under basal conditions, half of the ammonia generated by the kidney is excreted in urine, and the other half returns to the renal vein.^[12]^ At a physiological pH of 7.4, most (>98%) ammonia exists as NH4+ ions; however, diffusion across cell membranes occurs in the form of gaseous NH3.^[13]^ NH4+ relies on various transport channels, such as K+ ion channels and transporters or aquaporin-8 channels, to cross cell membranes and the blood-brain barrier.^[14]^ Glutamine synthetase activity in astrocytes protects the brain from high ammonia levels by converting ammonia to glutamine.^[15]^ Excess glutamine accumulation leads to morphological changes and dysfunction of astrocytes, which adversely affect neurological consequences.^[16]^ Thus, it is now recognized that an increase in blood ammonia concentration leads to higher levels of ammonia in the brain, which has a limited capacity for ammonia detoxification and is considered the main target of ammonia toxicity.^[17]^

Normally, low blood ammonia levels are maintained, which do not cause significant damage to the body.^[18]^ Plasma ammonia concentrations may vary with gestational age, and the reference intervals for ammonia among neonates, especially premature neonates, are not well defined. The published reference value for blood ammonia concentration in healthy newborns within 1 month is 21 to 95 μmol/L. This recommended threshold is also thought to fit very low birth weight infants (<1000 g).^[19]^ In addition, a single reference limit of 82 μmol/L for all newborns <1 week of age has been proposed.^[20]^ Increased ammonia production or insufficient ammonia detoxification can result in neonatal hyperammonemia.^[21]^ Hyperammonemia is usually defined as >100 µmol/L in neonates or ≥50 µmol/L in term infants, children and adolescents.^[22]^ When the blood ammonia level in a premature baby is above 150 µmol/L, investigation of possible inherited metabolic diseases is recommended.^[23]^ Neonatal hyperammonemia has been implicated in various pathological conditions (Table 1).

As shown in Table 1, THAN is an exclusive diagnosis that can only be made when other known causes of hyperammonemia are excluded.^[16,24–30]^ The mechanisms involved in THAN development are controversial. Fortunately, some clues have been derived from well-documented reports. Previous evidence suggests that the transient abnormality of the rate-limiting enzymes of the urea cycle or the temporary loss of urea-cycle enzyme function caused by delayed development of enzymes outside the urea cycle are responsible for THAN.^[31]^ Boys have seemingly had a higher incidence and worse clinical outcomes, which is considered another example of “male disadvantage” in perinatal morbidity and mortality.^[32]^ Hypoxic-ischemic damage secondary to perinatal asphyxia, which may result in decreased hepatic urea biosynthesis, is a commonly accepted factor associated with THAN.^[33]^ Another hypothesis is that shunting of the blood away from the portal circulation of the liver leads to a consequent decrease in the elimination of ammonia.^[34]^ Urinary concentrations of β-thromboglobulin and 11-dehydrothromboxane B2 are much higher in THAN infants, indicating that transient platelet activation in the portal system can also lead to THAN.^[35]^ Liver microcirculation is a unique vascular platform used to combat blood-borne infections.^[36]^ When severe systemic infection occurs, platelets adherent to the liver vasculature actively migrate to scan their microenvironment for bacteria; hence, microvascular thrombosis is inevitable.^[37]^ Therefore, it can be proposed that occlusion in the portal system due to secondary thrombosis triggered by platelet chemotaxis in the blood flow to the liver may be a pathological insult causing THAN in neonates with infection. This may explain the simultaneous occurrence of sepsis and THAN in newborns. THAN is an exclusion diagnosis for other known disorders and was based on almost five case reports in 1978.^[6]^ Most cases of THAN were reported in the 1970s and the 1980s and have been reported less frequently in recent years.^[38]^ These years of underdeveloped gene sequencing may explain why THAN has not yet been revealed. An example of a possible cause of THAN unraveled in recent years is the genetic condition of carbon hydrase Va deficiency, first described in 2014.^[39]^

**Table 1:** The causes of hypermmonemia.^[16,24–30]^

Inherited disorders	Acquired disorders
*Enzyme defects of the urea cycle*	*Acute or chronic liver failure*
N-acetylglutamate synthase deficiency	*Portosystemic shunt*
Carbamoylphosphate synthetase 1 deficiency	*Infection*
Ornithine transcarbamylase deficiency	Coronavirus disease^[16]^
Argininosuccinate synthetase deficiency	Urease-positive bacteria (C. neoformans, Mycoplasma hominis) overgrowth^[26,27]^
Argininosuccinate lyase deficiency	Genito-urinary infection
Arginase 1 deficiency	*Nitrogen overload*
*Transporter and synthesis defects of the urea cycle*	Total parenteral nutrition
Hyperornithinemia hyperammonaemia-hypercitrullinuria syndrome	Renal disease
Citrin deficiency	Catabolic stimuli (trauma, steroids, chemotherapy)
Ornithine aminotransferase deficiency	Massive hemolysis
Carbonic anhydrase Va deficiency	Protein catabolism from starvation
*Organic acidemias*	Gastrointestinal hemorrhage
Methylmalonic aciduria	*Drugs*
Propionic aciduria	Valproate
Isovaleric aciduria	L-asparaginase
3-Hydoxy-3methyl glutaryl coenzyme A lyase deficiency	Ribavirin
*Fatty acid oxidation defects*	5-flurouracil
Medium-chain acyl-CoA dehydrogenase deficiency	Teprenone
Multiple acyl-CoA dehydrogenase deficiency	Inhibitors of carbonic anhydrases (like topiramate)^[28]^
Carnitine palmitoyltransferase II deficiency	Asparaginase
Carnitine-acylcarnitine translocase	Sunitinib^[29]^
*Mitochondrial energy production disorder*	Methamphetamine^[30]^
Mitochondrial complex V deficiency	*Nutrition*
Mitochondrial complex III deficiency	Imbalanced total parenteral nutrition
Combined oxidative phosphorylation deficiency 5	Nutritional carnitine deficiency
*Others*	*Transient hyperammonemia of the newborn*
Hyperinsulinism/hyperammonemia syndrome	
Delta1-pyrroline-5-carboxylate synthase deficiency	
Lysinuric protein intolerance	
Glutamine synthetase deficiency	
Phosphoenolpyruvate carboxykinase deficiency	
Pyruvate carboxylase deficiency	
Dihydrolipoamide dehydrogenase deficiency	
Serine active site-containing protein 1 deficiency	

## 3. Diagnosis of THAN

Ammonia is a highly neurotoxic substance, and routine testing of ammonia levels should be performed if neurological involvement is determined, including lethargy, progression to somnolence and later coma, vomiting, refusal to feed, hypothermia, and seizures in neonates. Usually,these symptoms appear when ammonia concentrations reach 300μmol/L or even higher (400–500 μmol/L).^[40]^ Furthermore, ammonia concentrations >200 μmol/L are associated with cerebral edema and increased mortality.^[41]^ When hyperammonemia is confirmed, a series of metabolic investigations should be implemented, including quantitative amino acids in the blood and urine, organic acid analysis in the urine, acylcarnitine profile in the blood, and enzymatic assays and molecular tests as soon as possible.^[42]^ It is important to differentiate THAN from inherited metabolic disorders (IMD) because effective early therapy can be achieved with a good prognosis.^[43]^ In neonates, when the ammonia concentration is greater than 150 μmol/L, the source of hyperammonemia must be determined if it is related to inherited metabolic diseases, such as urea cycle disorders (UCD), organic acids, and fatty acid oxidation defects.^[19]^ Neonates with UCD exhibit primary respiratory alkalosis (PH > 7.45, low partial pressure of carbon dioxide [PCO_2_]) and hyperammonemia crisis after the first 24 hours of life.^[44]^ Neonates with organic acids usually experience hyperammonemia accompanied by metabolic acidosis, elevated blood lactate levels (>3 mmol/L), and ketonuria within 2 to 7 days of birth.^[45]^ Neonates with fatty acid oxidation defects also suffer from neonatal hyperammonemia and a history of low or undetectable serum glucose.^[46]^ Ammonia measurement is strongly recommended for all conditions of acute or intermittent neurological deterioration or psychiatric illness, acute liver failure, and suspected intoxication.^[47]^ Recently, genetic testing has been strongly recommended to confirm the diagnosis and exclude IMD.^[48]^ Generally, biochemistry-first approaches, such as tandem mass spectrometry, are used to identify IMD for which immediate treatment is required. Over the last decade, next-generation sequencing (NGS) technology has brought molecular diagnosis to a new field. The NGS is a powerful tool early in the diagnostic which allowed for the sequencing of millions of genomes and exomes.^[49]^ Compared to biochemical detection, NGS has a higher and more accurate positive diagnostic rate, especially owing to the lack of an identifiable biochemical footprint.^[50]^ NGS has potential applications in the neonatal IMD screening of dried blood samples. Tandem mass spectrometry and genome sequencing are the primary complementary methods used to confirm the diagnosis of IMD. Enzyme activity analysis is a time- and labor-consuming assay that can be used when metabolite patterns and genetic tests yield unclear results.^[51]^

Although asymptomatic hyperammonemia is widespread, serum ammonia determination is not routinely performed in the neonatal intensive care unit, resulting in an underdiagnosis of THAN. The biochemical characteristics of THAN include a normal amino acid profile, normal enzymes of the organic aciduria and urea cycle, and normal excretion of orotic urinary acids.^[52]^ An algorithm for the differential diagnosis of hyperammonemia was provided. In particular, the differential diagnoses include sepsis, intracranial hemorrhage, severe asphyxia, or seizures of undetermined etiology. Many clinical manifestations, such as feeding difficulty, vomiting, irritability, convulsions, and encephalopathy, mimic sepsis, especially in newborns with sepsis.^[53]^ Clinicians must consider this disease in their differential diagnosis. Hyperammonemia should always be considered when evaluating neonatal sepsis, especially in settings with poor feeding, vomiting, lethargy, seizures, and encephalopathy. When sepsis, intracranial hemorrhage, or asphyxia cannot explain coma and seizures, serum ammonia monitoring is vital.^[54]^

It often takes a few days to obtain laboratory results. Differentiating THAN as early as possible from other causes of hyperammonemia is significant when determining whether to execute specific treatment or enforce palliative care because correct management of THAN can be expected to achieve excellent outcomes. There are some distinct clinical manifestations that distinguish patients with UCED from those with THAN, which appear in the neonatal period. THAN is more likely to occur in premature infants (≤36 weeks’ gestation) or infants with low birth weight (≤2500 g at birth), an earlier onset of coma (≤24 hours), and a higher blood ammonia level (1500 μmol).^[55]^ Sclerema neonatorum may be an early manifestation of THAN because of a common unknown factor. Although not a specific recommendation, the author suggests that more attention should be paid to blood ammonia levels in infants who develops sclerema.^[56]^ It is difficult to perform differential diagnoses of IMD and THAN based only on clinical manifestations. The aquation method proposed by Kolchina et al^[57]^ could be helpful for the differential diagnosis of IMD and THAN before obtaining genetic sequencing results. The sensitivity, specificity, and diagnostic efficiency of the model were 87.5%, specificity −83.3%, diagnostic efficiency −85.0%.

## 4. Management of THAN

Although severe THAN has a more violent onset, rapid and timely treatment can lead to a good prognosis. An investigation by Yoshino et al^[58]^ showed that the survival rate of neonates with severe transient hyperammonemia was good, reaching more than 70%, whereas 53.9% of survivors did not develop any neurological sequelae. The duration of coma, peak ammonia levels, and significantly elevated intracranial pressure are correlated with the prognosis of hyperammonemic syndrome.^[59]^ When ammonia levels are higher than 200 μmol/L, the risk of death increases, and infants whose ammonia levels exceeded 1000 μmol/L, hyperammonaemic coma lasting >3 days, or increased intracranial pressure indicate a poorer prognosis.^[60,61]^ Recently, a noninvasive multimodal neuroimaging technique was used to predict long-term outcomes.^[62]^ As there is no efficient neuroprotective therapy for neonates with hyperammonemic coma, the rapid initiation of treatment is more important than the identification of the etiology.^[63,64]^ Prompt management of hyperammonemia in newborns is important to achieve successful neurodevelopmental outcomes.^[65]^ Treatment of the first episode of acute hyperammonemic encephalopathy, particularly in newborns, is similar regardless of the underlying diagnosis.^[66]^ Early measures must be taken to downregulate blood ammonia levels, including nutrition management, drug therapy, and/or supportive treatment.

### 4.1. Nutrition retention

The central issue in nutrition management is preventing catabolism by providing adequate caloric intake (carbohydrates and lipids).^[67]^ In particular, protein intake must be temporarily interrupted and intravenous glucose and lipids should be supplemented. To promote anabolism, energy intake should be approximately 120% of the age-adjusted requirements. Minimum energy intake of 100 kcal/kg/d was provided. Parenteral glucose intake should reach 8 to 10 mg/kg/min and lipid intake should begin from 0.5 g/kg, up to 3 g/kg daily.^[68]^ To promote anabolism and glucose utilization, insulin with 0.01 to 0.02 U/kg/h may be added to glucose solution with appropriate electrolytes (Na+, K+) to maintain blood glucose levels between 5.6 and 11.2 mmol/L.^[68,69]^ When the blood ammonia level drops below 100 μmol/L, proteins can be reintroduced within 48 hours because excessive protein restriction may lead to the catabolism of endogenous proteins.

### 4.2. Drug therapy

Drug therapy for THAN involves the delivery of substrates that may be deficient in metabolite scavengers (MS). The former, L-carnitine, hydroxy-cobalamin, biotin, and arginine, and the latter refer to sodium benzoate, sodium phenylbutyrate, and N-carbamyl-glutamate.^[70]^ Early administration of MS should be performed for the treatment of suspected hyperammonemia, a metabolic disease of intoxication, since it may avoid supportive therapy and the associated adverse effects. There are no standard-dose guidelines for the treatment of THAN, we only can refer to guidelines for UCDs. For THAN, medication can be administered with reference to the recommended drugs and doses for undiagnosed patients, including sodium benzoate, sodium phenylbutyrate/sodium phenylacetate, L-arginine, and N-carbamyl glutamate. Sodium benzoate is preferred in patients with mild symptoms. If hyperammonemia is not relieved or deteriorates, sodium phenylbutyrate/sodium phenylacetate should be added. In severely ill patients, sodium benzoate and sodium phenylbutyrate/sodium phenylacetate can be directly used in combination.^[71]^ Attention should be paid when prescribing intravenous medications because sodium benzoate is associated with severe toxicity in the central nervous system when administered at a dose higher than the recommended dose and arginine HCl is associated with hyperchloremic acidosis. The maximum daily doses of sodium benzoate and arginine should not exceed 12 g/d. Sodium benzoate, phenylbutyrate, or phenylacetate are usually used in combination with supplementation with the amino acids l-citrulline or l-arginine. Arginine supplementation is commonly used for IMD and asymptomatic hyperammonemia; however, it does not seem to affect plasma ammonium levels in symptomatic THAN.^[72]^ N-Carbylglutamate is a prospective medicine for the treatment of hyperammonemia due to its ability to increase ureagenesis and restart the urea cycle, thus reducing the need for or duration of peritoneal dialysis and hemodialysis.^[73]^

### 4.3. Supportive therapy

Supportive therapy remains a top priority as the clinical outcome of THAN goes hand in hand with the severity and duration of the hyperammonemia state and is highly dependent on the rapid elimination of excessive plasma ammonia. Treatment with MS combined with supportive therapy achieves in a more rapid normalization of ammonia levels than treatment with MS alone. Supportive therapy should be performed in neonates with the blood ammonia levels ranges from 250 to 500 μmol/L, a condition of which was previously not well controlled 4 hours after the start of drug therapy or as soon as possible in the presence of neurologic deterioration, regardless of plasma ammonia levels.^[74]^ Ammonia removal depends mainly on concentration gradient diffusion, which can be accomplished using peritoneal dialysis (PD), hemodialysis (HD), or continuous renal replacement therapy (CRRT). PD is less effective in the acute setting than CRRT or HD. HD is highly efficient for ammonia extraction, but its use in infants can cause severe technical and hemodynamic complications, and ammonia levels tend to rebound after HD.^[75]^ CRRT is preferred for neonates with hyperammonemia who often experience hemodynamic instability,^[76]^ and the rebound effect of plasma ammonia is not observed in CRRT-treated THAN.^[77]^ CRRT can provide suitable solute clearance of ammonia during continuous venovenous hemodialysis (CVVHD) or continuous venovenous hemodiafiltration (CVVHDF). CVVHD and CVVHDF are recommended as first-line treatments for ammonia detoxification in newborns as first-line treatment.^[78]^ CVVHDF exhibited an excellent ammonia-scavenging effect and was well-tolerated in newborns and infants. It has been proposed that successful CRRT requires adequate catheter performance and dialysate flow.^[79]^ In patients with a high level of ammonia, it is necessary to increase the median ultrafiltration rate above 4000 mL/h/1.73 m^2^ to achieve a good prognosis.^[80]^ Picca et al^[81]^ provided a typical CRRT prescription in Italy. When patients have blood ammonia levels > 1000 μmol/L, high-dose CRRT with Qb 30 to 50 mL/min, aiming at (dialysis fluid flow rate)/(blood flow rate) > 1.5, or HD may be used as the initial therapy.^[82]^ It is difficult to establish vascular access lines and maintain fluid balance in neonates with such a large circuit volume, which is a major obstacle in CRRT. Fortunately, new devices for CRRT in babies have been developed, such as the Cardio-Renal Pediatric Dialysis Emergency Machine and Newcastle infant dialysis and ultrafiltration system.^[77,83]^ Extracorporeal membrane oxygenation (ECMO) has proven to be a platform for the rapid removal of serum ammonia.^[84]^ HD or CRRT combined with ECMO can be used in neonates with life-threatening elevation of ammonia levels who are hemodynamically unstable.^[85]^ Coagulopathy may often occur when ECMO/HD is used and bleeding complications may occur less commonly.^[85]^ An increased risk of substantial cerebrovascular events has also been observed in neonates with ECMO-assisted CVVHD.^[86]^ PD is recommended only when other modalities of kidney supportive therapy are unavailable. Notwithstanding the higher efficiency of CVVHDF compared to PD, there seems to be no significant difference in the mortality rates of neonates.^[87]^ For infants with THAN, previous case reports have shown that exchange transfusion has some effect, although it is not as effective as HD or CRRT.^[38,68,88]^ Exchange transfusion is not recommended because it causes catabolism.^[89]^ If centers have less experience with extracorporeal detoxification or no conditions for extracorporeal detoxification, patients with a hyperammonemic crisis should be transferred to a specialist center without delay. Dialysis is usually continued until the plasma ammonium level is steadily less than 100 μmol/L.^[90]^

It is better to restart feeding during dialysis to evaluate the restoration of hyperammonemia before stopping dialysis, and enteral feeding should be restarted as soon as possible. In addition, mild systemic hypothermia appears to be effective in hyperammonemia coma because reduced temperatures make the enzyme less active, which in turn reduces ammonia production and leaves enough time for the removal of ammonia from the blood, but more studies are needed to determine its effectiveness.^[91]^

## 5. New developments for THAN treatment

During hyperammonemia, ammonia-induced depletion of liver alpha-ketoglutarate and its consequent inhibition of the mechanistic target of rapamycin kinase complex 1 (MTORC1) results in autophagy induction.^[92]^ It has been proposed that hepatic autophagy potentiates ammonia detoxification by providing key urea cycle intermediates (aspartate, acetyl-CoA, glutamate) and ATP. Hyperammonemia induces hepatic autophagy through α-ketoglutarate-dependent inhibition of MTORC1 as a consequence of intrahepatic α-KG depletion. Thus, hepatic gene transfer of the master regulator of autophagy transcription factor EB or treatment with the autophagy enhancers rapamycin and Tat-Beclin-1 can enhance autophagy to treat hyperammonemia.^[93]^

## 6. Conclusion

Although THAN has been known for decades, early differential diagnosis of THAN from other forms of hyperammonemia remains inadequate. Infants exhibit feeding difficulties, seizures, lethargy, or coma, which are conditions that are also caused by infection or other diseases; thus, THAN may be overlooked. However, there is no doubt that the management of premature infants should be improved, and blood ammonia monitoring should be included in the urgent diagnostic workup, at least when symptoms of neurological deterioration or psychiatric illness, acute liver failure, suspected intoxication, or in the differential diagnosis of neonatal sepsis. It is equally obvious that despite several drugs and dialysis methods for small pediatric patients have been introduced, there is an ongoing need for research on optimal modalities and dosing aimed at treating THAN. Several etiological mechanisms for THAN have been proposed, but a putative explanation for this rare entity remains to be explored.

## Author contributions

All the authors participated in the conception, design, analysis, interpretation, writing, revision, and approval of this article.

**Conceptualization:** Jun Zhao, Qie Guo.

**Data curation:** Beibei Ni, Jun Zhao, Qie Guo.

**Funding acquisition:** Qie Guo.

**Resources:** Miao Qin.

**Supervision:** Miao Qin.

**Validation:** Jun Zhao.

**Writing – original draft:** Beibei Ni.

**Writing – review & editing:** Jun Zhao, Qie Guo.
